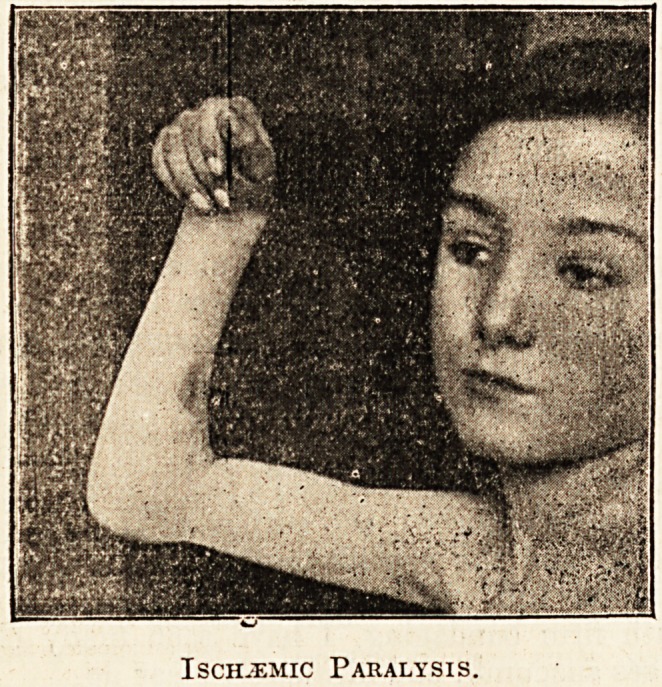# A Case of Ischæmic Paralysis

**Published:** 1908-01-25

**Authors:** Purves Stewart


### Notes on Current Neurology.

### A CASE OF ISCHÆMIC PARALYSIS.

By PURVES STEWART, M.D., F.R.C.P.

A few weeks ago a little boy, ten years of age, presented himself at the Royal National Orthopædic Hospital with the following history. Ten months ago he fell and sustained a fracture in the neighbourhood of the right elbow. He was taken to a hospital where the limb was put up in splints. The splints extended from the middle of the upper arm to the middle of the hand. A month later, when the splints were removed, he found himself unable to move his fingers properly, extension of the fingers being especially impaired. At the same time it was noticed that he had a small swelling in the flexor aspect of

Ischæmic Paralysis.

the forearm in its upper third. This swelling has since gradually subsided, leaving a depression in its place. The impairment in the movements of the fingers and hand has persisted, in spite of assiduous treatment by massage and electricity.

On examination of the affected limb, there is no obvious muscular atrophy. Sensation is perfect to all stimuli. There is a small indented area at the junction of the upper and middle thirds of the forearm in the site of the former swelling. All the muscles of the forearm feel abnormally hard and firm. No movement is absolutely lost, but there is marked limitation of all movements of the hand and fingers, extension being particularly impaired. The accompanying photograph (kindly taken by the house-surgeon, Mr. H. D. Davis) shows the maximum amount of voluntary extension of the wrist and fingers. The movements of the thumb are free in all directions. All the muscles of the forearm react to faradism, though less briskly than on the healthy side. There is also marked diminution of galvanic excitability, but no alteration in the polar reactions.

It will be noticed in this case that there is no actual paralysis of any individual muscle or group of muscles. All the muscles can be voluntarily innervated, but from want of extensibility the flexor muscles cannot be relaxed sufficiently to allow of the fingers being straightened even passively. The absence of sensory changes and the presence of faradic excitability with normal galvanic polar reactions excludes a paralytic lesion of any of the nerve-trunks, while the hard, almost gristly feel of the muscles, together with the posture of the hand and the history of the case is characteristic of v. Volkmann's ischæmic paralysis.

This is a somewhat rare affection which results from too tight application of bandages and splints, whereby the blood-supply of the subjacent muscles is interfered with. At first there is swelling and pain in the hand, and the forearm muscles become swollen from œdematous effusion. Later they become shrunken, abnormally hard from interstitial fibrosis, and gradually undergo contracture. The flexor muscles being normally more powerful than the extensors, the contracture is at the expense of the weaker muscles. Hence the hand becomes semi-flexed, and it is impossible to straighten completely the fingers or wrist, whether actively or passively. Treatment, as a rule, is of little avail, once the muscles have become infiltrated with fibrous tissue. Massage and warm arm-baths sometimes benefit these cases. In addition, I propose to try the effect of rubbing in an ointment containing thiosinamine.

## Figures and Tables

**Figure f1:**